# A Potential Tool for Clinicians; Evaluating a Computer-Led Dietary Assessment Method in Overweight and Obese Women during Weight Loss

**DOI:** 10.3390/nu9030218

**Published:** 2017-03-01

**Authors:** Adrianne M. Widaman, Nancy L. Keim, Dustin J. Burnett, Beverly Miller, Megan G. Witbracht, Keith F. Widaman, Kevin D. Laugero

**Affiliations:** 1 Department of Nutrition, Food Science and Packaging, San José State University, San José, CA 95192, USA; adrianne.widaman@sjsu.edu; 2 Nutritional Biology, University of California, Davis, CA 95616, USA; nancy.keim@ars.usda.gov; 3 United States Department of Agriculture, Agricultural Research Service, Western Human Nutrition Research Center, Davis, CA 95616, USA; dustin.burnett@ars.usda.gov (D.J.B.); bevmiller@ucdavis.edu (B.M.); 4 Institute for Memory Impairments and Neurological Disorders, University of California, Irvine, CA 92697, USA; mwitbrac@uci.edu; 5 Graduate School of Education, University of California, Riverside, CA 92521, USA; keith.widaman@ucr.edu

**Keywords:** weight management, dietary assessment, 24 h recall, weight loss programs, ASA24, obesity, women

## Abstract

Many Americans are attempting to lose weight with the help of healthcare professionals. Clinicians can improve weight loss results by using technology. Accurate dietary assessment is crucial to effective weight loss. The aim of this study was to validate a computer-led dietary assessment method in overweight/obese women. Known dietary intake was compared to Automated Self-Administered 24-h recall (ASA24) reported intake in women (*n* = 45), 19–50 years, with body mass index of 27–39.9 kg/m^2^. Participants received nutrition education and reduced body weight by 4%–10%. Participants completed one unannounced dietary recall and their responses were compared to actual intake. Accuracy of the recall and characteristics of respondent error were measured using linear and logistic regression. Energy was underreported by 5% with no difference for most nutrients except carbohydrates, vitamin B12, vitamin C, selenium, calcium and vitamin D (*p* = 0.002, *p* < 0.0001, *p* = 0.022, *p* = 0.010, *p* = 0.008 and *p* = 0.001 respectively). Overall, ASA24 is a valid dietary assessment tool in overweight/obese women participating in a weight loss program. The automated features eliminate the need for clinicians to be trained, to administer, or to analyze dietary intake. Computer-led dietary assessment tools should be considered as part of clinician-supervised weight loss programs.

## 1. Introduction 

Obesity is an increasing public health issue and is associated with decreased quality of life and increased morbidity [1]. According to recent data, two out of three American adults are overweight or obese [2]. Annual spending by medical, Medicaid, and private payers on weight and obesity-related health problems is estimated at $147 billion, with the non-inpatient portion costing $44 billion [3]. Seventy-six percent of Americans responding to a Gallup survey have wanted at some point to lose weight [4], with 49% of adults currently wanting to lose weight [5]. Thirty-seven percent of the women surveyed have tried to lose weight up to 10 different times [4]. Clinician-supervised weight loss programs led by either a medical doctor, nurse, nurse practitioner, physician’s assistant, registered dietitian, and/or a psychologist can assist people struggling to lose weight [6]. One study found that 42% of obese patients were advised on weight loss by a health-care professional [7].

Technology has been incorporated successfully for the treatment of patients who are overweight and obese [8]. A systematic review of the primary care setting suggested interventions incorporating technology assisted patients with weight-loss and control [8]. The American Heart Association (AHA) recommends that clinicians assess dietary intake as part of weight management care in ambulatory settings, and that accurate assessment of eating behavior is necessary even in the busy clinical setting [9].

Evaluating what a person truly eats and assigning nutritional value to the content of their diet is extremely difficult. Assessing dietary intake in overweight and obese populations is especially challenging. A disparity in misreporting exists, with obese respondents under-reporting significantly more often and to a greater degree than their normal weight counterparts. Doubly-labeled water studies suggest participants using 24-h recall methods under-report energy intake by 10%–25%, and under-reporting is increased further by 6.5% in people with Body Mass Indexes (BMI) of 30 compared to 25 kg/m^2^ [10]. Based on estimated energy requirements, one study found obese women under-reported energy intake by an average of 591 kcals per day as compared to women of normal weight and overweight women (178 kcal, 289 kcals respectively) [11]. Several studies have demonstrated that overweight and obese participants have a higher rate of under-reporting compared to leaner participants [10,11,12,13]. Obese subjects have been shown to under-report high carbohydrate and sugar-rich foods [14,15]. Therefore, it is crucial to further evaluate dietary assessment tools in overweight and obese participants. Accurate nutrient intake data will assist clinicians in identifying and targeting patients individualized nutrition needs to increase weight loss [9].

Multiple 24-h recalls have been found to be the most accurate dietary assessment method with the least systematic bias [16,17]. With the 24-h recall, a participant is asked to recall all foods and drinks consumed over the previous day from midnight to midnight. Traditionally, a single 24-h recall is administered in person by trained interviewers [18] and takes 20–30 min. Unfortunately, many clinicians do not have time or staff for 24-h recalls [9]. The Automated Self-Administered 24-h Recall (ASA24) is a dietary assessment tool designed as an alternative to the standard interview-administered 24-h recall [18]. ASA24 is designed for respondents to complete on their own [18], prior to seeing their clinician, thus freeing time during the in-person visit. In addition, this technology-based assessment tool may decrease social desirability bias and may be preferred by younger respondents [19]. The primary goal of any dietary assessment method is to estimate usual dietary intake. However regardless of administration method, no single 24-h recall can capture overall usual energy and nutrient intake. Nonetheless, the use of a validated 24-h recall method is an important advancement in weight loss/weight maintenance counseling because it can capture an estimate of dietary intake to aid clinicians in developing individualized, patient-centered interventions. 

ASA24 is modeled after the Automated Multiple Pass Method (AMPM) and relies on computer-generated prompts to gather retrospective dietary intake from respondents [18]. Participants are prompted to recall dietary intake at each meal or snack [20]. Details are collected on all foods and drinks reported, and participants find items by searching by name or food group [21]. Images of differing servings and images of household measurements are provided to aid in estimating portion size [22]. ASA24 nutritional information is generated from the United States Department of Agricultures Food and Nutrient Database for Dietary Studies (FNDDS).

ASA24 was developed using extensive formative research [20,21,22]. In addition, the performance of the computer-administered ASA24 was compared to the interviewer-administered AMPM method without any major differences being identified [19,23]. Although the ASA24 has been suggested as a tool for clinicians [18], it has not been validated in a population participating in a weight-loss program. Further evaluation of computer assisted, dietary assessment tools in overweight and obese populations is needed. Additional studies would contribute to current knowledge of the validity and error associated with online dietary assessment methods. This would allow the translation of this tool which is currently used mainly for research studies by clinicians for weight management. 

In the context of known dietary intake, the aim of this study was to validate the use of a computer-based dietary assessment method in overweight and obese women who received weight-loss counseling. In addition, the possible causes of dietary misreporting were investigated by examining characteristics of foods not reported and portion size estimates. 

## 2. Material and Methods

### 2.1. Study Design

This study was a secondary objective of a more comprehensive parent study (registered as Clinical Trials number NCT01410838). The parent study consisted of three phases: (1) weight loss; (2) transition; and (3) weight maintenance. The ASA24 validation study reported here was conducted after the weight-loss phase, during the transition phase. For the parent study, recruitment of Northern California residents in Sacramento and Yolo County took place between 2011 and 2014. The weight-loss portion of the study was a nine-week program that included nutrition education and one-on-one nutrition counseling sessions. The training was coupled with the participants completing food records that were reviewed weekly by the study nutritionist. During this weight-loss phase, participants were briefly introduced to the ASA24 program (Automated Self-Administered 24 h Recall (ASA24)-2011. Bethesda, Rockville, MD, USA: National Cancer Institute).

In the two-week transition phase that followed weight loss, participants were provided a controlled diet, and all plate waste was measured. During this transition, one day was chosen where participants completed an unannounced ASA24 recall. This study was approved by the University of California, Davis, Institutional Review Board, and all participants provided written informed consent. 

### 2.2. Participants

Participants aged 19–50 years were recruited throughout Sacramento and Yolo County. Women with a BMI between 27 and 39.9 kg/m^2^ were included. Women were excluded if they were hypertensive, diabetic, vegetarian, pregnant, or if they used tobacco. Two hundred and sixteen women completed a phone screen. One hundred nineteen participants were enrolled in the weight-loss phase of the parent study. Enrolled participants completed a nine-week, weight-loss phase. Forty-seven of the participants lost 4%–10% of their original body weight. These forty-seven continued to the transition phase of the study. Forty-five completed the ASA24 recall and constitute the sample for this study.

### 2.3. Anthropometrics/Body Composition/Demographics

Body weight was measured after the weight-loss phase and reported to the nearest 0.1 kg using a Tanita BWB-627A Class III electronic scale with subjects wearing lightweight surgical scrubs. Height was measured to the nearest 0.1 cm using a wall-mounted, Ayrton Standiometer Model S100 (Ayrton Corporation, Prior Lake, MN, USA). Body Mass Index (BMI) was calculated as kg/m^2^. Participants self-reported race and years of education. 

### 2.4. Actual Dietary Intake

Over one full day, participants were provided a morning meal, a midday meal, an afternoon snack, and an evening meal. The diet was composed of 55% carbohydrate, 28% fat, and 17% protein. All items were prepared and measured by the Metabolic Kitchen and Human Feeding Laboratory (MK&HFL) at the Western Human Nutrition Research Center. The morning meal, midday meal, and afternoon snack were consumed at the Research Center. The dinner meal was packaged and sent home with the volunteer so it was not consumed onsite. The MK&HFL used a packout method that has been used successfully in other controlled feeding studies [24]. Participants were instructed to return all leftovers in the provided containers. All plate waste from the inpatient meals and the packout dinners was measured by the MK&HFL. The participants were familiar with the foods and beverages offered as they had already received 7–16 days of food and beverages from the MK&HFL at this point in the study. In addition, each item was visibly labeled with the food type and quantity. Participants were instructed to consume all and only the study foods provided by the MK&HFL. Known intake was measured by calculating the difference between what was provided and any food or beverage that remained. The actual intake was entered into the ASA24 system by two trained nutritionists following a standardized order entry process. The energy and nutrient information was generated by the ASA24 program.

### 2.5. Reported Dietary Intake

On one of the test days where participants spent the full day in the Metabolic Research Unit, they received verbal instructions to complete an ASA24 recall of the previous day. The request for the recall was unannounced. The foods and beverages selected by the participants using ASA24 were considered “reported intake”. The participants selected the type of each food/beverage item, portion size, and the time the meal was consumed. 

### 2.6. Classification of Items

When compared to actual intake, reported intake was coded as a true match, close match, far match, exclusion, or false inclusion. For example, if the respondent actually consumed “chicken breast”, an exact match would be “chicken breast”, a close match would “chicken, any type” and a far match would be “poultry (type not specified)”. If the food or beverage was not reported, the item was coded as an exclusion. For reader clarity, exclusions will also be referred to as “foods not reported”. Finally, if the item was reported but not actually given, it was labeled a “false inclusion”. This coding system was used in another ASA24 validation study [23]. Items were grouped into dairy, added fat, fruit, grain, nut, protein, added sugar, and vegetable groups. Food/beverage items were also coded as main meal items or additions to the main items. Coding was done separately by a Registered Dietitian and the staff nutritionist and compared; differences were agreed upon. Portion size was not considered during coding. 

### 2.7. Portion Size

The gram values of reported intake were subtracted from actual grams eaten and the difference was divided by the actual intake. This resulted in a “+” percentage for an overestimation of portion size, a “0” for an exact report of portion size, or a “−” percentage for an underestimation of portion size. 

### 2.8. Statistical Methods

Statistical analyses were performed using SAS, version 9.2 (SAS Institute, Inc., Cary, NC, USA) and SPSS version 22 (SPSS, Inc., Armonk, NY, USA). 

#### 2.8.1. Exclusion

For descriptive purposes, the mean proportion of items not reported was calculated by food group. The differences in proportions of items excluded at each meal time point were measured using repeated measures analysis of variance (ANOVA) (α = 0.05). 

To determine the association between food group and the likelihood of an item not being reported (i.e., being excluded from the participants’ recall), excluded foods were coded (1) and exact, close, and far matched foods coded (0). Logistic regressions with effect-coded predictors representing food groups were used to obtain measures of the odds ratio of a food group being excluded during the recall. Using effect coding of food groups allowed the odds ratio of exclusion for each food group to be compared to the grand mean level of exclusion. If the odds ratio for a given food group was significantly greater than (or less than) one, the odds of excluding instances of this food group were significantly greater than (or less than) the odds of excluding food items on average. The model was adjusted for non-independence of individual respondent’s exclusion rates, or clustering at the participant level, by including effects-coded fixed factors for participants. Therefore, the model’s intercept, when exponentiated, represents the average likelihood of not reporting an item. The parameter estimates for food groups are regression weights in the logistic model for predicting exclusion. The probability of exclusion was determined by exponentiating the sum of the intercept parameter estimate and the individual food group parameter estimate. 

The same method described above was used to determine the likelihood of a main food item not be reported as compared to an additional item. The effect of whether an item was a main or addition was then separated by food group and the above model was repeated.

#### 2.8.2. Portion Size

Only foods with exactly matching ASA24 food codes were evaluated. “Closely” matched foods were not included because they often included combination foods and ASA24 portion size options were not identical. Overestimates of reported portions were truncated at 300% (which affected only 6 out of 445 items across participants). For descriptive purposes, mean true and reported portion sizes in grams were calculated. The proportion of reported portion sizes within 25% of truth was described. Linear regression with effect-coded predictors was used to measure the main effects of food group and eating occasion on the reported portion size difference from the actual portion size. After testing for main effects, we estimated a full-rank regression model without an intercept so the mean effect of the food group could be directly estimated.

#### 2.8.3. Energy/Nutrients

The total energy, micronutrient, and macronutrient intake for each participant was derived from the participants’ reported dietary intake. ASA24 nutrition analysis software converts food/beverage items into energy and nutrient values based on the Food and Nutrient Database (FNDDS). The mean, standard deviation, and difference from actual intake were calculated using the actual and reported intake of each nutrient. Linear regression was used to compare the difference of reported intake to actual intake. The intercept was removed allowing the differences to be compared to zero. A score of zero represents exact agreement between the actual and reported nutrient values. The model was adjusted for BMI, age, and percent weight loss with little effect, therefore these control variables were removed from the analysis.

## 3. Results

### 3.1. Participants

One hundred and nineteen participants were screened and enrolled in the weight-loss phase. Forty-seven of these participants lost 4%–10% of their initial weight and continued the study in the transition phase. Forty-five women averaging 37.4 ± standard deviation (SD) 9.7 years of age completed the controlled feeding of the transition phase, including the ASA 24-h recall, and were included in this analysis. The women included had lost an average of 5.3% ± SD 1.7 of their initial weight, and their average BMI was 31.1 kg/m^2^ ± 3.6 at the time of the recall. Twenty-five participants reported their race/ethnicity as White, eleven as Hispanic, six as Asian, and three as Black. The participants’ mean years of schooling was 15.0 years (SD 1.6 years) which in the United States is the equivalent of some college. 

### 3.2. Classification of Food/Beverage Type

Sixty-eight percent of the items were main items, and 32% of the items were items that were additions to the main food and beverage items. Main items were four times less likely than additions to not be reported (Odds Ratio (OR) 0.28, 95% Confidence Interval (CI): 0.20–0.39). When the items were categorized by food group, the items in the animal protein group were only main items. The nuts, added fat and added sugar groups were only categorized as addition items. However, the dairy, fruit, vegetables and grains group had both main and addition items. Within the dairy, fruit and vegetable groups, whether the item was a main item or addition had no significant effect on the likelihood of not reporting the item during the recall (OR 0.38, 95% CI: 0.10–1.46; OR 1.40, 95% CI: 0.15–12.88; OR 1.86, 95% CI 0.29–11.83, respectively). Grains that were main items were extremely unlikely to be forgotten (OR 0.05, 95% CI: 0.02–0.14).

Seventy-nine percent of items reported by participants were exact or close matches to the actual items consumed with an additional 5% being far matches. The mean proportion of items not reported (exclusions) was 16% (Table 1). A description of the items excluded by food group is reported in Table 2. The average proportions of items excluded during breakfast, lunch, snack, and dinner were similar (15.6% ± 13.8, 16.9 ± 19.0, 13.6 ± 26.6 and 15.5 ± 18.3, respectively). No significant effect of the type of meal on the proportion of items excluded was found (*p* = 0.913). 

To quantify how strongly food group type was associated with exclusion rate, logistic regression analyses were performed and this showed that food group was significantly related to exclusion rate (*χ*^2^ (7) = 43.22, *p* < 0.0001). Results of the logistic regression analyses are shown in Table 3, which provides parameter estimates (with standard errors), the modeled probability of exclusion, and the odds ratio (with 95% CI) for exclusion relative to average exclusion rate. Only three of the eight food groups had odds ratios that differed significantly from the average exclusion rate. The odds of nuts/seeds being excluded from the recall was 80% higher than the grand mean (OR 1.80, 95% CI: 1.18–2.76). The odds of failing to report added sugar was 400% higher than average (OR 4.06, 95% CI: 2.28–7.22). Conversely, the odds of not reporting an animal protein food was lower than average (OR 0.29, 95% CI: 0.14–0.61). 

### 3.3. Portion Size

For the recalled items that matched exactly with actual items, portion size estimates were examined. For descriptive purposes, mean true and reported intake are reported in Table 4. Reported portion size estimates within 25% of true portion size range from 34.1% for protein foods to 57.6% for nuts/seeds (Table 4). All food groups except grains trended toward being overestimated, with fruit, nuts/seeds, animal protein, dairy, and vegetables being significantly different (*p* = 0.01, 0.02, 0.003, < 0.001, and < 0.001, respectively) than actual portion sizes (Table 4). Portion sizes of vegetables were overestimated by an average 48% and represented 36% of the falsely included items. The portion sizes of items recalled for the dinner meal were underestimated by an average of 20% (Table 4). 

### 3.4. Nutrients

The differences between reported nutrients and actual nutrients are described in Table 5. A negative difference represents under-reporting and a positive difference represents over-reporting by the participant using ASA24. Most nutrient values generated by the ASA24 software based on the reported dietary intake did not differ significantly from actual intake values. Energy was under-reported by an average of 115 kilocalories. Total carbohydrates, vitamin C, selenium, and vitamin B12 were significantly under-reported (*p* = 0.002, 0.02, 0.01, and < 0.0001, respectively). Calcium and vitamin D consumption were significantly over-reported (*p* = 0.008 and 0.001, respectively). Compared to other nutrients measured, the magnitude of difference between reported Vitamin B12 intake and actual intake was the largest (−57.3%).

## 4. Discussion

### 4.1. Performance of Computerized Recall

In the current sample of overweight and obese women, ASA24 performed well, with under-reporting of energy at 5% of actual energy intake as compared to under-reports of 15%–50% with other dietary assessment methods evaluated in the general population [10,11,12,13]. This computer-assisted tool was shown to accurately measure energy intake in overweight and obese women who had participated in a weight-loss program. 

In this study, the proportions of matched (“exact”, “close”, and “far”) recalled items to actual items were very similar to a previous validation study of ASA24 by Kirkpatrick et al. [23]. In a population of men and women with various body weights (80%, and 84%, respectively). Mismatched food items may explain the under-reporting of vitamin C and vitamin B12 intake in this study. Another ASA24 validation study found vitamin C values differed when participants entered foods eaten into a nutrient database instead of the investigators [25]. The under-reporting of vitamin B12 intake may be an artifact of this study design. In this study, a snack that contained a vitamin B12-fortified ready-to-eat cereal (RTEC) was often misclassified by participants with an alternative snack not containing RTEC being selected. 

### 4.2. Possible Causes of Misreporting

One review of the etiologies of under-reporting in dietary assessments described the behavior of excluding items as either intentional or unintentional [26]. Social desirability bias is suspected to be present in free-living diet assessments and has been suggested as a cause of intentional misreporting [26]. However, in our study, it is unlikely that social desirability led to intentional exclusion because the meals and snacks were not selected by participants. It is more likely that excluded food items were genuinely forgotten [26]. In this study, main meal items were four times less likely than small additions to the meal to be excluded during the recall. Specifically, it was very unlikely for main grain items to be excluded. Other studies have found that excluded foods were most commonly small components or additions to the meal [23,26] versus the main dish items. 

Although ASA24 was designed for ease of use and engagement [18,20], participant burnout and/or difficulty finding items in the ASA24 search function could both have contributed to the exclusion rate of 16%. Respondents may exclude foods during an ASA24 recall due to poor memory or lack of attention to dietary intake [26]. Clinicians using ASA24 should realize that foods not reported by respondents may have different effects on dietary intake results depending on whether food groups or nutrients are being reported. For example, a patient forgetting to recall added sugar and added fat additions will appear to be eating less energy, and the clinicians may miss the opportunity to target these areas for weight loss interventions. Therefore in conjunction with the ASA24 recall, additional probing by the clinician to determine intake of energy dense additions would be suggested. In the current study, calorically dense foods (nuts/seeds and added sugars) not reported may account for the under-reporting of energy and carbohydrates. Other studies comparing actual intake to recorded intake have previously identified under-reporting of added sugars in obese women [15]. 

In addition, portion size estimation is difficult because it involves multiple cognitive abilities [22,27]. ASA24 prompts respondents to identify from memory the correct portion size by using images and household units as prompts [18]. The results from this study found estimates of portion size tended to lie within an accurate range for added fats, grains, and added sugars. When looking at the meal level of portion size data, respondents were able to accurately estimate the size of the breakfast and lunch meals. Dairy and vegetables were significantly overestimated. The portion size of certain food groups may be more difficult to estimate. In addition, the typical amorphous and soft shapes of food items in the dairy and vegetable groups (i.e., shredded cheese, scoopof broccoli) may be more difficult for the participant to estimate. Kirkpatrick et al. results suggested that portion size estimates of food items with amorphous/soft shapes tended to be overestimated from true portion size intake using ASA24. Only eight percent of amorphous/soft food portion estimates fell within 10% of truth [28]. In a study validating the Automated Multiple-Pass Method (on which the ASA24 was based), obese women overestimated their macronutrient intake [29]. A study comparing investigator versus participant-entered dietary intake suggested inaccurate portion size estimates by ASA24 users may have led to nutrient differences [25]. In this study, nutrients found in dairy, such as calcium and vitamin D, were reported at a level significantly higher than actually consumed (9.4% and 16.1%, respectively). A study measuring the ability of female nutrition students to estimate portion size also found the portion size of milk to be overestimated [30]. Another ASA24 validation study found that Vitamin D levels were over-reported [23] and were significantly higher than the AMPM measurement [19]. Our results found that vegetable portion size was overestimated by 48% of actual intake. Other studies have also shown that portion size estimates of vegetables using different recall methods are inaccurate [30,31]. The average underestimation of portion size at dinner maybe related to the meal itself having a larger portion of protein and vegetable food items. Participant fatigue, as this was the last eating occasion entered, and/or packout meal service may have attributed to the estimate error. 

Twenty-four hour dietary recalls were used to prompt the participants to recall intake over the previous 24-h period. In our study, the longest duration between a meal and the recall was the previous breakfast and the shortest was the previous dinner. In children, it has been found that the closer the recall time is to the meal consumed the more accurate the report [32]. However, we found no difference in exclusion rate due to the duration of the recall time.

#### Strengths/Limitations of Study

This study is not without limitations. The sample size is small which may limit our findings. We studied a distinctive group of overweight and obese women who had success losing weight. In addition, the participants were familiar with the foods provided which may limit the results generalizability. However, this study is unique in that it validates a dietary assessment tool in overweight/obese women undergoing a weight loss program. To our knowledge, evaluation of any dietary assessment tool in this population (*n* = 45) has not been done. 

### 4.3. Use in Practice

By eliminating an in-person interviewer, the goal of ASA24 is to increase the feasibility and decrease interviewer time needed to capture high quality food, beverage, and supplement data [18]. A previous study conducted to determine the primary care providers’ “wish list” for online weight loss programs found that monitoring tools that were not dependent on manual entry of calorie data were desirable [33]. ASA24 fits this description. Many validated dietary assessment tools are often seen as impractical for busy clinicians because the administration and analysis is time-consuming and requires training [9]. This study suggests that ASA24 is a valid dietary assessment tool in an overweight/obese population participating in a weight management program. The automated features of ASA24 eliminate the need for clinicians to be trained, to administer, or to analyze 24-h recalls. Clinicians can prompt patients via email to independently complete recalls prior to visits. This would allow the clinician to avoid spending time collecting and analyzing food records during short in-person visits. 

In most cases, ASA24 will provide an accurate assessment of energy and nutrient intake in a 24-h period. Multiple dietary recalls including weekend days would provide the clinician with a better estimate of the patient’s usual intake [10,13,17]. Depending on the type of nutrition-related problem of the patient, the ASA24 results should be verified with other measures. For example, in specific cases where vitamin C, vitamin D, calcium, and possibly vitamin B12 are of concern, a clinician could target nutrient-specific food groups during the clinical visit and verify possible deficiencies based on the full clinical picture (i.e., physical exam, medical history, laboratory values and medication usage) of the patient.

## 5. Conclusions

In obese women involved in a weight-loss program, ASA24 appears to be a valid dietary assessment tool. Respondents were able to recall and match the majority of items consumed. Energy and carbohydrate intake were under-reported by 5% and 10%, respectively. Excluded foods tended to be additions to main meals (nuts and added sugars), and portion size was slightly over-estimated, especially for vegetables. ASA24 could be a useful tool to determine dietary intake for clinicians working with overweight and obese, female clients. 

## Figures and Tables

**Table 1:** Mean proportions of Automated Self-Administered 24-h recall (ASA24) reported types of food/beverage coded as exact matches, close matches, far matches, excluded or falsely included items as compared to actually consumed items by overweight and obese female respondents.

Reported Items Code ^a^	Mean Proportion (%)	Standard Deviation (%)
Exact match	52.3	15.2
Close match	26.5	9.8
Far match	5.1	4.8
All matches combined	84.0	10.7
Excluded	16.0	10.7
Falsely included	3.5	4.5

^a^ code based on type of item reported. Portion size not considered.

**Table 2:** Descriptive statistics of items not reported during ASA24 recall by food group in overweight and obese female respondents.

Food Group	Number of Items	Mean Proportion of Items Not Reported (%)	Standard Deviation(%)
Dairy	207	12.1	32.7
Added fat	90	15.6	36.4
Fruit	93	21.5	41.3
Grain	225	15.1	35.9
Nuts/seeds	117	25.6	43.9
Animal protein	91	5.5	22.9
Added sugars	47	45.5	50.0
Vegetables	150	12.0	32.6

**Table 3:** Odds of food groups not being reported (excluded) during an ASA24 recall in overweight and obese female respondents.

Food Group	Parameter Estimate (SE)	Modeled Probability of Exclusion	OR (95% CI) ^a^
Intercept	−1.70 (0.12)	0.18	N/A
Dairy	−0.39 (0.21)	0.14	0.68 (0.45, 1.01)
Added fat	−0.06 (0.27)	0.17	0.94 (0.56, 1.60)
Fruit	0.32 (0.24)	0.25	1.37 (0.85, 2.21)
Grain	−0.17 (0.19)	0.15	0.85 (0.59, 1.23)
Nuts/seeds	0.59 (0.22) *	0.33	1.80 (1.18, 2.76) ^b^
Animal protein	−1.25 (0.38) **	0.05	0.29 (0.14, 0.61) ^c^
Added sugar	1.40 (0.29) **	0.74	4.06 (2.28, 7.22) ^b^
Vegetables	−0.44 (0.24)	0.12	0.65 (0.41, 1.03)

* *p* value < 0.01. ** *p* value < 0.001. ^a^ Calculated using logistic regression and adjusted for individual exclusion rate. Results are significant if the confidence interval does not include an odds ratio of 1. ^b^ Indicates a food group whose odds of being excluded was higher than the grand mean. ^c^ Indicates a food group whose odds of being excluded was lower than the grand mean. SE, Standard Error; OR, Odds Ratio; CI, Confidence Interval; N/A, Not applicable.

**Table 4:** Mean true and reported portion sizes, proportion of reported within 25% of truth, and results of regression analysis of reported percentage portion size over- or underestimate of actual portion size consumed, by food group and meal type (*n* = 445 items).

Food Group/Meal	Mean Amount Eaten g (SE)	Mean Amount Reported g (SE)	Proportion of Reported Portion Sizes within 25% of Truth	Average Over/Underestimate of Portion Size ^a^ % (SE)	*p*-Value
Dairy	170.7 (11.0)	208.6 (12.8)	50.0%	27.7 (1.0)	<0.001
Added fat	10.6 (1.2)	10.8 (1.1)	42.9%	31.1 (17.4)	0.075
Fruit	77.5 (8.0)	86.7 (8.1)	54.1%	21.7 (8.6)	0.011
Grain	53.8 (4.3)	47.4 (3.5)	49.6%	0.3 (6.1)	0.96
Nuts/seeds	12.0 (0.9)	14.8 (1.3)	57.6%	26.7 (11.6)	0.021
Animal protein	51.0 (4.3)	61.3 (9.3)	34.1%	33.1 (11.2)	0.003
Added sugars	16.6 (2.5)	15.3 (1.7)	37.5%	28.8 (23.1)	0.212
Vegetables	50.5 (7.7)	55.5 (5.5)	38.5%	48.2 (8.5)	<0.001
Breakfast	85.1 (9.2)	92.9 (8.8)	55.3%	7.6 (4.9)	0.123
Lunch	66.8 (4.5)	76.4 (5.9)	36.8%	2.9 (4.7)	0.536
Dinner	79.0 (11.2)	75.5 (11.8)	43.4%	−20.2 (7.1)	0.005

^a^ A positive value represents the average percentage reported overestimation compared to actual portion size. A negative value represents the average percentage reported underestimation compared to actual portion size. Calculated using linear regression, α = 0.05.

**Table 5:** Difference between actual and reported energy and nutrient intake using ASA24.

Nutrient	Mean Actual Intake	Mean Reported Intake	Mean Difference ^a^ (CI)	Magnitude of Difference ^b^ (%)	*p*-Value ^c^
Energy (kcal)	2202.8	2087.9	−114.9 (−230.3, 0.46)	−5.22	0.051
Total Protein (g)	90.4	91.6	1.2 (−4.1, 6.5)	1.31	0.65
Total Fat (g)	65.4	66.1	0.7 (−4.9, 6.3)	1.10	0.80
Total Carbohydrate (g)	326.8	294.4	−32.3 (−51.7, −12.9)	−9.89	**0.002**
Total Dietary Fiber (g)	29.3	27.3	−1.9 (−4.0, 0.1)	−6.61	0.06
Calcium (mg)	1254.3	1371.7	117.4 (32.1, 202.7)	9.36	**0.008**
Iron (mg)	20.3	20.3	0.0 (−1.2, 1.2)	−0.03	0.99
Magnesium (mg)	425.9	427.9	1.9 (−26.9, 30.8)	0.45	0.89
Phosphorus (mg)	1753.7	1773.8	20.1 (−83.1, 123.3)	1.14	0.70
Copper (mg)	1.6	1.6	0.1 (0.0, 0.2)	5.97	0.19
Potassium (mg)	3223.9	3311.8	87.9 (−105.8, 281.6)	2.73	0.37
Sodium (mg)	3049.14	3262.31	213.2 (−4.2, 430.6)	6.99	0.054
Zinc (mg)	15.5	15.9	0.4 (−0.5,1.3)	2.54	0.40
Selenium (mcg)	117.9	108.3	−9.6 (−16.8, −2.4)	−8.15	**0.010**
Vitamin C (mg)	141.2	124.7	−16.5 (−31.2, −2.6)	−11.69	**0.022**
Dietary Folate Eq (mcg)	863.2	902.5	39.3 (−16.1, 94.6)	4.55	0.16
Vitamin B12 (mcg)	4.5	1.9	−2.6 (−2.9, −2.3)	−57.33	**<0.0001**
Total Saturated Fat (g)	19.5	21.0	1.5 (−0.2, 3.2)	7.65	0.09
Vitamin D (mcg)	5.1	6.0	0.8(0.4, 1.3)	16.08	**0.001**

^a^ Reported intake—actual intake; a negative value indicates under-reporting and positive value indicates over-reporting. ^b^ Magnitude of difference = (mean difference/mean of actual intake) × 100. ^c^ *p* values calculated using linear regression and represent the difference between actual and reported intake.
